# PROTOCOL: The impact of infrastructure on low income consumers' nutritious diet, women's economic empowerment, and gender equality in low‐and middle‐income countries: An evidence and gap map

**DOI:** 10.1002/cl2.1353

**Published:** 2023-09-05

**Authors:** Gloria Adobea Odei Obeng‐Amoako, Clarice Panyin Nyan, Joseph Clottey, Sheila Agyemang Oppong, Edward Kusi Asafo‐Agyei, Pacem Kotchofa, Charles Yaw Okyere, Solomon Zena Walelign, Takyiwaa Manuh, David Sarfo Ameyaw

**Affiliations:** ^1^ International Centre for Evaluation and Development Tema Accra Ghana; ^2^ Department of Agricultural Economics and Agribusiness University of Ghana Legon Ghana; ^3^ School of Economics University of Gondar Gondar Ethiopia

## Abstract

This is the protocol for an evidence and gap map. The objectives are as follows: this evidence and gap map (EGM) aims to identify, map, and provide an overview of the existing evidence and gaps on the impact of different types of physical infrastructure on various outcomes of low‐income consumers' nutritious diet, women's economic empowerment, and gender equality in low‐ and middle‐income countries. The specific objectives of the EGM are: (1) identify clusters of evidence that offer opportunities for evidence synthesis and (2) identify gaps in evidence where new studies, research, and evaluations are needed.

## 1 BACKGROUND

### 1.1 Introduction

#### 1.1.1 The problem, condition, or issue

Malnutrition remains a major global health challenge, especially among young children, despite the continued efforts of the international community to address it (Ahmed, 2012). Malnutrition refers to deficiencies or excesses in nutrient intake, imbalance of essential nutrients, or impaired nutrient utilization (WHO, 2022). In 2020, about 150 million children worldwide under the age of five were reported stunted, with almost 45 million wasted and 40 million overweight or obese (United, 2020a). Similarly, about 1.9 billion were overweight while 462 million of them were underweight (United, 2020b). Vulnerable social groups such as women, infants, children, and adolescents are at higher risk of malnutrition. Also, poor people are more likely to experience different forms of malnutrition, that is, undernutrition, micronutrient deficiencies, and obesity (United, 2020a) Malnutrition also raises healthcare costs while reducing productivity and slowing economic growth, which can perpetuate a cycle of poverty and ill‐health. However, according to (Marshak, 2021), malnutrition is mainly seasonal, particularly in African drylands. Seasonality in malnutrition involves variability in nutritional outcomes correlated with changes in climatical conditions such as temperature, rainfall, and vegetation affecting food production systems. Seasonal changes in these variables, mediated through livelihoods and institutions, can reinforce issues surrounding consistent intakes of healthy and nutritious food, food security, health, and driving malnutrition (Marshak, 2021; Young, 2020). Seasonality can be measured through year‐round fluctuations in prices and food availability affecting producers and consumers, particularly in South Asia (SA) and Africa.

Poor people's seasonal food accessibility, that is, year‐round food availability and affordability, is a multifaceted problem with few concrete solutions being deployed at scale. Hence, understanding factors influencing the agri‐food systems to enhance nutritious food supply and shape people's nutrition and social development is critical for geographically targeted policy interventions and trade‐offs associated with them. According to the WHO 2020, the nutrition needs of low‐income consumers (LICs), especially women and children, in low‐and middle‐income countries (LMICs) are often compromised due to poor infrastructure underpinning the agri‐food systems (WHO, 2020). Also, even where different types of infrastructure exist, their designs are not always gender‐inclusive, hampering women's and girls' access to food (Morgan, 2020). While gender equality implies equal rights, responsibilities, and opportunities for women, men, boys, and girls; women and girls are disproportionately affected by persistent gender inequalities when accessing infrastructure (United Nations Secretariat and United Nations Entity for Gender Equality and the Empowerment of Women, 2020). Women and girls are unable to access the basic services to support their upward social mobility and reduce the gender gap because most infrastructure in developing countries is underdeveloped and gender‐blind. Consequently, “gender‐blind infrastructure fails to consider the different roles, responsibilities, and particular needs of women, men, girls, and boys in a specific context and how this affects their ability to use or access infrastructure” (Morgan, 2020). Gender inclusivity must guide the design and uptake of future infrastructure, especially those related to agri‐food systems (Mohun, 2016; Morgan, 2020).

Shively (2017a) argues that well‐functioning infrastructure such as market centers and roads could foster gender‐inclusive socioeconomic development and mitigate the adverse effects of seasonality on the availability and price of nutritious food. Shively (2017b) found that investments in health, market infrastructure, agricultural innovations, and transport infrastructure positively correlate with child nutritional status in developing countries. Additionally, infrastructure is a game‐changer for development and enhances women's economic opportunities; because when more women get the chance to work, it makes their families, communities and countries wealthier (Mohun, 2016). Hence, well‐designed and gender‐inclusive investment in infrastructure can support meeting various SDGs, among which are SDGs 1, 2, 3, 5, 6, 7, and 8 (World, 2020). Nonetheless, to the best of our knowledge, no study has explored the extent of existing evidence and gap on the impact of infrastructure on Nutritious Diets, while also empowering women and promoting gender equality in LMICs. To address this gap, the International Centre for Evaluation and Development (ICED) is designing an evidence and gap map (EGM) to review all relevant evidence on links connecting some pertinent infrastructure and nutritious diet, women's economic empowerment (WEE), and gender equality for LICs in LMICs in sub‐Saharan Africa (SSA) and SA.

#### 1.1.2 Why it is important to develop the EGM

To our knowledge, there is no existing EGM on the impact of physical infrastructure on nutritious diets, WEE, and gender equality. There are some general maps for instance on transport in LMICs (Malhotra, 2021), but the EGM has a broader focus in terms of outcomes, narrowly focused on one type of infrastructure (i.e., distribution infrastructure—roads, rail, trams, and monorail, ports, shipping, and inland waterways, and air transport) and restricted focus quantitative studies. Hence the study doesn't have a specific, detailed focus to be used in this map.

Previous systematic reviews (see e.g., Nandi, 2021), in addition to impact evaluations (see e.g., Kihiu, 2021), have analyzed the effect of specific infrastructure on the outcome variables of interest (see also Bryan, 2022; Dinkelman, 2011; Mahmud, 2018; Passarelli, 2018; Stifel, 2017). This study is the first to provide comprehensive evidence and gap on the impact of a wider typology of infrastructure on nutrition, women empowerment, and gender equality in LMICs to inform research and evidence on the impact of infrastructure on nutritious diet, WEE, and gender equality. By doing so, it will inform the design and evidence‐informed decision‐making on infrastructure to achieve broader goals enunciated in the SDGs (2, 3, 5, and 10). Additionally, this study would provide evidence and gaps on the level of advancement of gender equality as set out in the Beijing Declaration and Platform for Action (UN Women, 1995).

### 1.2 Conceptual framework (the theory of change)

**Figure 1:**
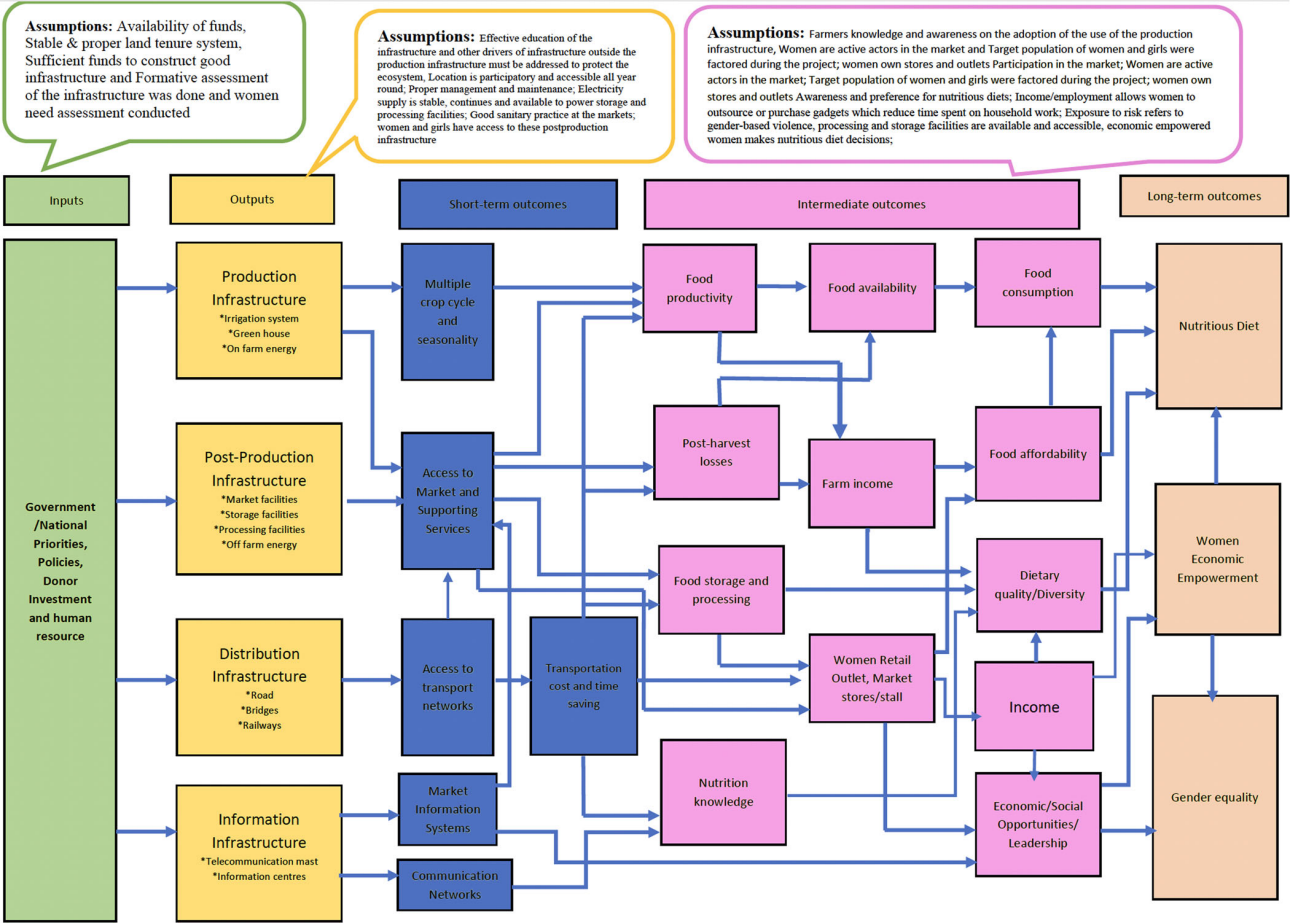
Conceptual framework for the theory of change.

The lack of well‐functioning infrastructure poses a substantial risk to rural livelihoods and threatens food and nutrition security; depriving millions of LICs of safe, nutritious, and affordable food (WHO, 2020), particularly women and girls. The conceptual framework of this EGM explains how the interventions mentioned above are hypothesized to affect the three outcomes of interest ‐ nutritious diets, WEE, and gender equality (Figure 1). The framework demonstrates how infrastructure typologies lead to either short or intermediate‐term outcomes, which eventually result in long‐term outcomes. Four infrastructure typologies are explained in this framework. They include; production infrastructure where interventions such as irrigation, greenhouse, and on‐farm energy are considered. The second infrastructure is post‐production infrastructure where interventions such as market facilities, processing facilities, storage facilities, and off‐farm energy were considered. The third infrastructure is distribution infrastructure where interventions such as roads, railways, and bridges were considered and finally, the fourth infrastructure is information infrastructure where interventions such as telecommunication masts, radio stations, and information centers were considered.

Production infrastructure which comprises interventions like irrigation systems, greenhouse and energy systems used for on‐farm activities impact all three outcomes through various channels. For instance, by using irrigation facilities, farmers can produce crops throughout the year reducing the effect of seasonality. Production infrastructure does not only enhance multiple crop cycles and reduce seasonality but also improves access to the market. These improve food productivity at the farm level. As a result of the improvement in food productivity at the farm level, the income of farmers is improved, and food becomes available at the farm gate as well as the market level. The availability of food enhances both individual and household food consumption eventually leading to nutritious diet. Income of farmers also enhances food affordability which also leads to nutritious diet. Production infrastructure also results in WEE and gender equality. The production infrastructure enhances the emergency of markets. Women take advantage of these markets to open retail stores where they can sell commodities. This serves as employment to these women and creates both social and economic opportunities for these women which eventually results in their empowerment as well as equality.

Post‐production infrastructure comprises of market, processing, storage facilities as well as energy systems used for off‐farm activities. An intervention like market facility leads to reduction in post‐harvest losses which enhances the income of the individual and the household as well as improves the availability of food at the market level. As a result of improvement in income, households can afford food which in the long‐term leads to nutritious diet. Similarly, with food being available as a result of reduction in post‐harvest loss, individuals or households enhance their consumption of food which also leads to nutritious diet in the long‐term. In SSA and SA most of the processing and storage facilities are operated or owned by women (Negash, 2015). Operation of these facilities leads to both social and economic opportunities for these women as well as employment. This in the long‐term leads to empowerment of these women as well as bridging the equality gap. Another channel where this post‐production infrastructure can lead to WEE and gender equality is through ownership and operating of retail markets or stores. As shown in the overall theory of change, due to the presence of storage facilities, food can be stored for future use. They can also be processed into another state other than the raw state from the farm. With this, women who are largely the operators or owners of small retail stores or outlets gain employment.

Distribution infrastructure enhances access to transportation networks which lead to reduction in transportation cost for producers in both the input and product market. Reduction in transportation cost of produce leads to a reduction in input prices which enhances food productivity. Food productivity will lead to food availability at the farm level as well as improvement of income of both the farmer and the household. This eventually led to nutritious diet in the long‐term. Also, with the reduction of transport cost, women are encouraged to establish market outlets and retails stores. This creates employment for these women, improve their income levels as well as enable them to take up social roles in the community. In the long‐term, the establishment of the road which is a distribution infrastructure intervention ensures that the women are empowered and bridged the gender equality gap. Investment in information infrastructure, like radio stations and information centers, improves information dissemination about nutrition. With enhanced knowledge of nutrition, households or individuals make informed decisions about that diet which eventually leads to nutritious diet.

## 2 OBJECTIVES

This EGM aims to identify, map, and provide an overview of the existing evidence and gaps on the impact of different types of physical infrastructure on various outcomes of LICs' nutritious diet, WEE, and gender equality in LMICs. The specific objectives of the EGM are:

- Identify clusters of evidence that offer opportunities for evidence synthesis.
- Identify gaps in evidence where new studies, research, and evaluations are needed.

## 3 METHODS

### 3.1 Defining an EGM

Several definitions of an EGM exist in the literature (e.g., Lum, 2011; Saran, 2018; Snilstveit, 2013). Following Saran (2018), we define an EGM as “a systematic visual presentation of the availability of relevant evidence of effects for a particular policy domain.” The evidence is identified by a search following a pre‐specified, published search protocol. Along with the map, the study will present a descriptive report that summarizes the evidence and gap for use by relevant stakeholders such as researchers, research commissioners, policymakers, and practitioners (Saran, 2018). We will not synthesize the results as done in systematic reviews (Potter, 2010). The proposed EGM will serve as a platform for a body of evidence on the relationship between infrastructure, nutritious diets, WEE, and gender equality. The EGM will be made accessible to its potential users.

### 3.2 Scope of EGM

We will apply the stipulated procedures and standards described in the Campbell Collaboration checklist and guidance for EGMs (White, 2018; White, 2020). The scope of this proposed EGM will be guided by the population, intervention, comparison, outcomes, and study designs (PICOS framework). It will cover studies that demonstrate the effect/impact of physical infrastructure—as a result of establishing or upgrading ‐ on nutritious diets, WEE, and gender equality (see Table 2 for the indicators for the outcomes). We do not look at the intersection between the three outcomes of interest. Evaluation and studies examining the effects of infrastructural interventions on the outcomes of interest using quantitative (i.e., experimental, quasi‐experimental, and non‐experimental), systematic reviews, and meta‐analyses, will be covered in this EGM. Unlike most other previous EGMs, we will also include studies with qualitative study designs. Similar to previous EGMs (e.g., CGIAR, 2021; Moore, 2021), this EGM will include studies published in the year 2000 and beyond for two main reasons. First, based on a preliminary search, we find out that most of the relevant studies are from 2000, and hence doing an unrestricted search would result in more studies that are not relevant, which increases the screening task to the team. Second, we are interested in the evidence and gap in the recent literature.

#### 3.2.1 Population

The population of interest for this EGM is LICs living in LMICs across SSA and SA regions. We define LICs as “individuals whose financial resources or income results in them being unable to obtain the goods and services needed for an “adequate” and “socially acceptable” standard of living” (Darley, 1985). We will exclude a study from the EGM if it was conducted in countries other than LMICs as classified by the World Bank in 2022. We define LMICs listed under SSA and SA with per capita Gross National Income (GNI) below USD12,695. This definition encompasses lower‐income countries (GNI less than USD1, 046), LMICs (GNI ranging from US$1046 to 4095), and upper‐middle‐income countries (GNI per capita between US$4096 and US$12,695) (World Bank, 2022). However, if a study conducted in the LMIC does not specifically focus on LICs but presents a general population that paper will be eligible for our study. Unless the authors state that the study population was urban rich then that paper will be illegible for the EGM. LMIC classification was not based on the year of intervention or previous country status to avoid complications. We used the LMIC classification by the World Bank as of 2022. We will use the World Bank's income classification in 2022 as the basis for deciding which countries are considered LMICs.

#### 3.2.2 Intervention

The intervention for this EGM is establishing new or upgrading existing **physical infrastructure**, which refers to establishing new or upgrading existing physical infrastructures that are relevant for agriculture, food production, marketing, and thus local development. While infrastructure can be broadly defined as the basic physical and organizational structures and facilities (e.g., markets, roads, power supplies, etc.) needed for a society or enterprise (Oxford, 2018; World, 2022), this EGM is limited in scope to the impact of the **physical presence** of infrastructures, not services provided by them. This EGM will focus on four types of infrastructure (see Table 1 for description): These are: (i.) production (such as irrigation systems and water wells); (ii.) post‐production (such as market centers and storage facilities); (iii.) distribution (such as transportation‐ roads, bridges); and (iv.) Information infrastructure (such as masts and radio stations). The choice of the categories is based on two considerations. First, the focus of the study is on the physical infrastructure mostly used by LICs whose welfare impacts could be quantified in developing countries, particularly in SSA. Second, these broad categories of physical infrastructure have been implemented by government and development organizations in improving welfare in developing countries to meet the needs of LIC as well as women and girls.

**Table 1:** Categories, sub‐categories, and intervention examples (infrastructure).

Categories of infrastructures	Definition	Sub‐category^a^
***Production infrastructure***	Facilities that are used in agricultural production or any other facilities that are in place to enhance agricultural productivity.	Irrigation systems, water wells, greenhouse, on‐farm power supply (energy)
***Post‐production infrastructure***	Facilities that are used for storing, processing, and marketing products as well as facilities that are used to ensure the supply of healthy foods in safe environments.	– Storage (warehouses and sheds, cold rooms, pack houses) – Processing (grain mills) – Market place (spaces, stalls, and lockups, provided with sanitary facilities (toilets) and childcare centers) – Off‐farm power supply (energy) – Others (Slaughterhouses, landing sites, livestock vaccination parks)
***Distribution infrastructure***	Facilities that are used for transporting inputs and products to ensure that LICs, including farmers, have access to markets and increased access to nutritious and diversified foods.	– Roads, – Railways, – Bridges
***Information infrastructure***	Facilities used to support the dissemination of information on good agricultural practices, nutritional knowledge, child feeding practices, weather information, markets, and credit, etc.	Information centers, radio stations, provision of telecommunication masts to facilitate and enhance communication

^a^ See Supporting Information: Appendix 1 for definitions of some of sub‐categories of infrastructure considered in the evidence and gap map.

#### 3.2.3 Comparison

This EGM does not specify a comparison group. However, studies with comparison groups or designs that estimate impact using data from treated and untreated observations (such as difference‐in‐differences) will be included.

#### 3.2.4 Outcomes

The EGM focuses on three outcomes: nutritious diets, WEE, and gender equality. The categories and sub‐categories are presented in Table 2.

**Table 2:** Definition of outcomes.

Outcome domains	Sub‐outcomes	Examples of indicators
***Nutritious diets***	Food availability: Farm level	Quantity of food produced per area (kg/hectare)
	Food Availability: Market level food	Availability of nutritious foods (fruits, vegetables, dairy, eggs, meat, fish, legumes, nuts) (e.g., specific metrics could be market volumes sold or transported or aggregated, number of market stands selling these items, etc.), Market Level Dietary Score (MLDS): Number of foods or food groups available in local markets at a given point in time
	Food accessibility: Affordability	– The volatility of food prices – Income variation in food access – Market Level Dietary Score (MLDS): Number of foods or food groups available in local markets at a given point in time. – Weather seasonality indexes in agriculture – Women empowerment in nutrition index (WENI)
	Food accessibility: Consumption and experiences	Per capita dietary energy supply (DES)kcal/per capita/day, Food Consumption Score (FCS), Food Groups Consumed, food variety score, Food Insecurity Experience Scale (FIES), HOusehold Food Insecurity Access Scale (HFIAS), Household Hunger Scale (HHS)
	Diet quality^a^ (nutrient adequacy) individual dietary diversity	Individual dietary diversity scores (DDS), Minimum Acceptable Diet (MAD) for infant/child (6–23 months), Minimum Dietary Diversity (MDD) for infant/child (6–23 months), Minimum Dietary Diversity for Women between 15–49 years (MDD‐W), Mean adequacy ratio (MAR): measures an individual's intake of the nutrient
	Diet quality^a^ (nutrient adequacy) household dietary diversity	Household dietary diversity scores (HDDS)
	Socioeconomic and cultural dimensions of foods	Women empowerment in nutrition index (WENI), food preferences
***Women's Economic Empowerment***	Agricultural production	Input in production decisions, autonomy in production
	Access and control over productive resources	*Ownership of assets; purchase, sale, or transfer of assets: access to, and decisions on credit*
	Income	*Control over the use of income*
	Time allocation	*– Workload, Leisure*
	Leadership	*Group Member, Speaking in Public*
	Women Empowerment in Agriculture	*Women Empowerment in Agriculture Index* (WEAI)^b^
	Gender Parity Index (GPI)^c^	*Women's achievements in the 5 domains* (5DE),^d^ *relative to the men in their households* e
***Gender Equality***	Economic Opportunities and Outcomes	*Women and men have equal opportunities in agricultural production systems, value chains, markets, resources (GAGP)*,^f^ *and entrepreneurship*
	Social outcomes	*Discriminatory and unequal social, cultural and gender norms change to enable women and men to participate equally in household and community institutions*
	Leadership, Agency, and Collective Action	– *Women's agency, leadership, and decision‐making are recognized and affirmed in the household and community* – *Women can engage in collective action to protect their interests*
	Reduced Exposure to Risk	*– Gender‐based violence (Cf. Pro‐WEAI* + *MI)‐ legislation and institutions exist to protect against GBV, and women are empowered to take action, leading to low and reduced rates of GBV* g

^a^ Dietary diversity represents qualitative measures of individual or household food consumption that reflect access to a variety of food groups and is used as a proxy for nutrient adequacy of the diet of individuals (INDDEX, 2015; INDDEX, 2018). We usually count 9 food groups listed as follows: (1) cereals, (2) starchy roots, (3) legumes, (4) vegetables and fruits, (5) sugars, preserves, and syrups, (6) meat, fish, and eggs, (7) milk and milk products, (8) fats and oils, and (9) beverages.

^b^This is measured using the Women's Empowerment in Agriculture Index (WEAI) (Alkire, 2013). WEAI is an aggregate index and is composed of two sub‐indexes‐ the five domains of empowerment (5DE), and the gender parity index (GPI). 5 Domains of empowerment (5DE), specified in the sub‐outcomes (Alkire, 2013). The overall WEAI is a weighted average of 5DE and GPI, with weights of 0.9 and 0.1, respectively (Malapit, 2015).

^c^ The GPI measures women's achievements in the five domains compared with the men in their households. All these indexes have values ranging from 0 to 1, where higher values reflect greater empowerment. Households are classified as having gender parity if either the woman is empowered (her empowerment score is 80% or higher), or her score is greater than or equal to the empowerment score of the male decision‐maker in her household. It is only calculated for dual‐headed households.

^d^ The 5DE is constructed from individual level empowerment scores which reflect each person's achievements in the five sub‐outcomes (see Table 2) and measured by the 10 indicators with their corresponding weights. Each indicator measures whether an individual has surpassed a given threshold, or has adequate achievement, with respect to each indicator.

^e^ As in Malapit (2015) (op cit.), note, gender parity is not equal to gender equality.

^f^ Gender Assets Gap Project. Cf. (Deere, 2013).

^g^ According to Nationen (2015), unsafe market spaces, transport, and public spaces expose women workers and traders to violence and limit their economic opportunities.

#### 3.2.5 Study designs

This EGM targets both published and unpublished studies, reports, and reviews that had applied mixed methods, quantitative and/or qualitative methods with the following study designs:

- Effectiveness studies (impact evaluations studies that used Randomized Controlled Trials (RCTs) and quasi‐experimental studies).
- Non‐experimental quantitative studies.
- Process evaluations.
- Summative evaluations.
- Qualitative studies.
- Systematic reviews and meta‐analysis of eligible studies.

#### 3.2.6 How we plan to handle adverse outcomes

Adverse effects of infrastructural intervention reported in the eligible papers will be captured in the EGM to avoid one‐sided summaries of evidence (White, 2020). To accommodate this, we labeled our outcome as neutral so that studies that report either of the outcomes are coded in the relevant subdomains. Thus, if a paper reports desirable outcomes as well as undesirable outcomes for the specific subdomain, we will code the paper in the subdomain under consideration. If the paper reports a desirable outcome in one subdomain and an undesirable outcome in another subdomain, the paper will be coded in both subdomains.

### 3.3 Criteria for including and excluding studies

We will assess the eligibility of the included studies (i.e., published, and unpublished) based on the population, intervention, outcome, and study design (PICOS) defined for this EGM (White et al., 2018).

#### 3.3.1 Inclusion criteria

##### Type of studies

This EGM will include all kinds of studies that applied all kinds of study designs, such as quantitative, qualitative, or mixed methods. Eligible study designs will consist of studies that assess infrastructure's impact on nutritious diets, WEE, and gender equality and will include, but not be limited to the following: effectiveness studies (impact evaluations/experimental studies); modeling studies (only if the model was based on primary data and not hypothetical values) process evaluations; summative evaluations; qualitative assessments; and analytical frameworks. Having a wide range of study designs in this EGM will provide the opportunity (i) to include all potential studies given the paucity of literature on the topic of interest and (ii) to assess the extent of evidence across a spectrum of study designs. Eligible qualitative studies should have a clear description of research methods (such as e.g., exploratory, narrative, deductive, inductive, ethnographic, and grounded theory approaches) and a detailed description of data collection techniques (focus group discussion, in‐depth interviews, key informants, sample size). Systematic reviews and meta‐analyses of the eligible primary studies will be included in the EGM. We will consider peer‐reviewed papers, pre‐prints, reports, and other documents (e.g., discussion papers, working papers).

##### Geographical location

Eligible studies for the EGM must have targeted LICs living in LMICs across SSA and SA regions. We will also include studies that considered both eligible and ineligible participants (population) irrespective of whether they reported their data and results separately. These studies will be coded for the eligible population during the data extraction. Nevertheless, we will enrich the rationale of the EGM when necessary, with relevant studies conducted in other geographic areas as in CGIAR (2021).

##### Timeframe

This EGM will include studies published/reported in the years 2000 and onwards.

##### Language

Studies written in English will be included in the EGM. This is because most of the researchers of the current study are only English‐speaking meaning that considering studies that are not written in English will require additional resources for translation (Neimann, 2018).

#### 3.3.2 Exclusion criteria

Studies published in languages other than English and conducted in locations other than LMICs in SSA and SA will be excluded from this EGM. Furthermore, this EGM will not include studies in areas other than LICs. Studies conducted in the period before the year 2000 will be excluded. Also, studies on infrastructure that are relatively less prevalent in LMIC and less accessible to the LICs in the LMIC, such as airports and harbors will be excluded from the EGM. Studies that do not assess the causal linkage of one or more of the infrastructural interventions on a nutritious diet, WEE, and gender equality as study outcomes will not be included in the EGM.

### 3.4 Search strategy, screening, and coding

#### 3.4.1 Search strategy

With the support of an information specialist, we will adopt a search strategy that will identify all potential published and unpublished studies for this EGM. This will help minimize publication bias. We will search for potential studies from three relevant scientific and academic databases: CAB Abstract, Greenfile Ebsco, and Medline Pubmed. Also, we will search for additional potential papers using OpenAlex in Eppi‐reviewer software (Thomas, 2010). OpenAlex is an index of hundreds of millions of interconnected entities across the global research system (Priem, 2022). We will also do a search on databases such as Agriculture, Nutrition and Health (ANH) Academy for proceedings of conferences and abstracts.

We will also search for studies and papers/reports from websites of relevant organizations, such as the World Bank, International Fund for Agricultural Development (IFAD), United Nations Women, United Nations Food and Agriculture Organization (FAO), International Food Policy Research Institute (IFPRI) and the Alliance for a Green Revolution in Africa (AGRA) and IMMANA grant database (Supporting Information: Appendix 2). We will also search the organizational websites and repositories of CGIAR group, IFAD, IIED, AgriProFocus, Bill and Melinda Gate Foundation, Donor Committee for Enterprise Development, Swiss Agency for Development and Cooperation, Department for International Development (DFID), Innovation Poverty Action (IPA) and The Abdul Latif Jameel Poverty Action Lab (J‐PAL), USAID Development Experience Clearinghouse and United Nations Office for Project Services (UNOPS).

We will use the backward‐track citations approach to check for additional primary studies, reviews, and meta‐analyses that may not be identified in our search but are eligible for our EGM. We will particularly look out for authors who are most cited in papers in the subject matter field for additional information on completed or ongoing studies to ensure that we capture all important evidence in the EGM. We will also look for ongoing and completed studies in trials, as well as reviews, and EGMs in registries or repositories. Including ongoing studies will provide information on emerging future studies and hence inform future plans for updating the EGM. However, we will restrict a timeframe of not more than 5 years within which a new study was registered as a decision rule to avoid including uncompleted studies which are long overdue.

We will glean additional studies in gray literature, evaluation reports, and academic theses. Existing relevant EGMs conducted by organizations such as Campbell Collaboration and 3ie will be a valuable resource for potential studies we could include in our EGM. All the eligible papers will be converted into Research Information System (RIS) and uploaded into EPPI‐Reviewer software (Thomas, 2010). We will keep a log of literature search activities for reporting purposes.

#### 3.4.2 Search terms

Based on the PICOS framework, we will generate search terms for retrieving published studies or reports from electronic databases. As indicated earlier, we intend to include studies with any study designs, including qualitative studies and process evaluation. Hence, the search terms for this EGM will not be restricted to any study design. Rather, we will use study designs as filters in displaying the EGM. Supporting Information: Appendix 2 shows the list of organizational websites for gray literature search. While Supporting Information: Appendix 3 shows examples of the search terms we will use to search for the studies/papers.

#### 3.4.3 Screening and coding

**Figure 2:**
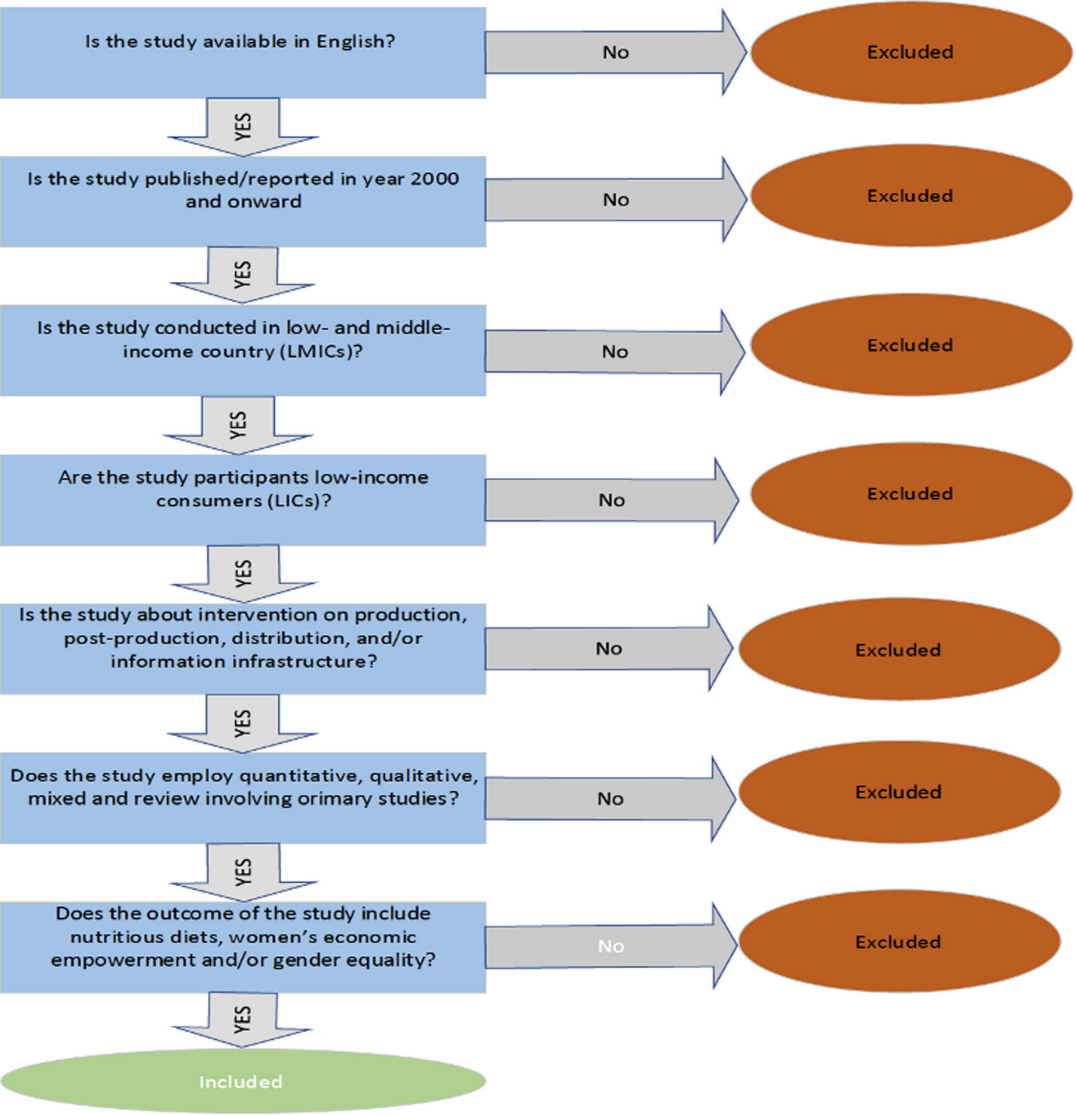
Screening tool showing inclusion and exclusion criteria.

Before screening the potential studies or papers, all duplicates will be identified and removed using the EPPI‐Reviewer software. To make screening of the papers faster, we will adopt a machine learning model to aid the ordering of the identified papers by relevance/priority based on the eligibility criteria for the proposed EGM review. Eligibility screening of published and unpublished studies or papers for the EGM will involve two stages. First, the studies will be screened for title and abstract based on the eligibility criteria (inclusion and exclusion criteria) as shown in Figure 2. Second, a full‐text screening will be undertaken for the studies to be included based on title and abstract screening. All studies will be screened by two reviewers independently. In cases of discrepancies in the decision to include or exclude a paper for the EGM, the reviewers will discuss and if they disagree, a third reviewer will conduct the screening independently to resolve the differences. Then studies that are included in the EGM will be coded and extracted. We will use EPPI‐Reviewer for coding, data management, and analysis (Thomas, 2010). Each study or paper will be coded in EPPI‐Reviewer based on a predefined form and duplicate data extracted for comparison (refer to Supporting Information: Appendix 4: Coding form).

#### 3.4.4 Data cleaning

The coded data set in the EPPI‐Reviewer will be cleaned before analysis. We will use univariate analysis to examine any out‐of‐range codes. We will investigate the reasons for the outliers and address them. The team will thoroughly review random samples of the data. All missing papers will be identified, and if possible, corresponding authors will be contacted them to ensure that all relevant literature is included in the EGM (White, 2020).

#### 3.4.5 Data analysis plan

The unit of analysis of the EGM will be a study, meaning that each item entered in the EPPI‐Reviewer will represent a study. A study is defined as an analysis of a unique sample, which may include multiple time points for the same sample (White, 2018). As in other EGMs, when multiple publications are identified on a single study, for instance, a working paper and a published paper, we will consider including the most recent open‐access publication in the EGM (Malhotra, 2021). Suppose a study reports on multiple interventions or outcomes or study designs, the study will be considered as one entry in the EGM but will be assigned codes for the interventions or outcomes, or study designs. Similarly, if a single study has multiple interventions with some being ineligible for our EGM, we will only consider the interventions relevant to our EGM.

However, primary studies appearing in a systematic review that qualify for our EGM will be considered as a unit of analysis and will be counted once.

Summary statistics such as percentages will be used to describe the distribution and characteristics of the studies in the EGM. The findings will be presented in graphs, tabulations, and cross‐tabulations of intervention versus outcomes categories, regions, and study design in a narrative report for the EGM. We will then summarize and synthesize the results to identify clusters of evidence and gaps. The utility of the EGM and any potential limitations identified will be discussed. EPPI‐Reviewer software will be used to generate descriptive statistics for the technical report and to create the EGM based on the data uploaded into the EPPI‐Reviewer (Thomas, 2010).

#### 3.4.6 Presentation of EGM

Like the EGM on transport by Malhotra (2021), our EGM will consist of a matrix with searchable filters. The interventions and outcomes are the two main dimensions of an EGM matrix. Through a consultative engagement with critical stakeholders (Campbell Collaboration and Bill and Melinda Gates Foundation), we will define the map framework: the row and heading titles and filters. We anticipate that the matrix will have intervention categories and subcategories as row headings against outcomes domains and subdomains as column headings. The matrix cells will contain the studies relevant to that intervention and outcome combination. The searchable filters will cover interventions, study design, study status (completed and ongoing), country and region, and location (Malhotra, 2021; White, 2018).

#### 3.4.7 Critical quality appraisal of studies

Appraising the quality of the primary studies will require the use of a checklist per the study design employed in each primary research, which is time‐consuming. Hence, given the tight deadline for delivering the EGM, we will not appraise the quality of the primary studies. We are not also appraising the quality of reviews included in the EGM as the number of reviews included in the EGM is very few. However, we will code and present the study designs employed by the eligible studies as filters for displaying the EGM (White, 2020).

### 3.5 Pilot study of EGM

Before creating our EGM, we pilot our search strategy, screening, and coding tools for the proposed EGM. In a pilot study, we will test and develop our search strategy in an iterative process. We will assess the suitability of the coding form with a small number (20–30) of the eligible studies for the EGM (Albers, 2019; White, 2020). The EGM framework, that is, the row and column headings for the interventins and outcomes with their sub‐categories, search strategy, and the coding form will be revised, refined, and re‐defined based on the findings of the pilot study after each round of the pilot exercise (White, 2020). While we will consider refining the labels of the categories of interventions, outcomes as well as filters, which we have not previously thought of in the iterative pilot exercises, we will be cautious not to make our map cumbersome and not user‐friendly (White, 2020). We will conduct a trial of the EGM to assess the practicality of EGM presentation and features as part of the pilot exercise. The trial EGM will be shared with our stakeholders (development practitioners, academicians, policymakers, Campbell Collaboration, and Bill and Melinda Gates Foundation) to seek their feedback on the EGM features such as the searchable filters, and labels for the row and column headers. We will include the final search strategy and coding forms in our EGM protocol. The principal investigator (PI) and the same team of researchers dedicated to EGM development will conduct both the pilot exercise as well as the actual EGM (White, 2020).

### 3.6 Stakeholder engagement

We will consult development practitioners, academicians, and policymakers familiar with the subject matter of the scope, design, and production of the EGM. We will work closely with the Campbell Collaboration from the conceptualization, design, and production of the EGM. We will also consult the Bill and Melinda Gates Foundation and other similar stakeholders (e.g., MasterCard Foundation) for their input at every stage of the EGM development. We will work with relevant stakeholders to disseminate the final EGM amongst relevant organizations, institutions, and networks. The EGM will be a public good; it will be published in an open‐access journal so that it is accessible to everyone. We anticipate that the findings of our EGM will inform future decision‐making on research and funding priorities on infrastructure, nutritious diets, WEE, and gender equality.

### 3.7 Plans to update EGM

The proposed EGM will be updated every two years when sufficient further studies and resources become available.

### 3.8 Sources of technical and financial support

This EGM will be developed in collaboration with Campbell Collaboration. Campbell Collaboration has a track record of experience in EGM production and capacity building for EGM. Campbell Collaboration will provide technical backstopping, while the Bill and Melinda Gates Foundation will give technical input from the funder's perspectives and financial support to produce the proposed EGM.

## CONTRIBUTIONS OF AUTHORS

**Content expertise**:

- David Sarfo Ameyaw, the CEO/President of ICED, is the PI for the proposed EGM and a content expert in food security and infrastructural projects in developing countries. He has over 30 years of experience in leadership and practical experience in international development, monitoring and evaluation, learning, research, and EGM.
- Takyiwaa Manuh, the gender specialist and a distinguished University Professor, Emerita, is a subject matter expert in women's empowerment and gender equality. She brings to the team several years of experience, skills, and knowledge of women's empowerment and gender equality in shaping the content of the EGM.
- Charles Yaw Okyere is a lecturer at the Department of Agricultural Economics and Agribusiness, University of Ghana, Legon, and a Research Associate at ICED. Charles Yaw Okyere holds a Doctor of Agricultural Sciences (Dr. Agr.) degree from the University of Bonn, Germany. His research interest is to generate rigorous evidence for policy making through applying behavioral, experimental, and quasi‐experimental economic techniques to agriculture, health, education, and welfare.
- Solomon Zena Walelign, the research director of ICED, is an experienced environmental and resource economist with expertise in forest sciences. He is also a consultant at the World Bank and an Adjunct Assistant Professor at the University of Gondar. He brings research experience from Nepal, Ethiopia, Kenya, and Tanzania to the team.
- Pacem Kotchofa, the nutrition specialist at ICED, has expertise in international development and agricultural economics.
- Gloria Odei Obeng‐Amoako, a nutrition specialist at ICED and experienced in research, has been working in the field of nutrition, public health, and epidemiology for over a decade.

**EGM methods expertise**

- David Sarfo Ameyaw, the CEO/President of ICED and the principal investigator (PI) of this EGM. He has several years of experience in international development, monitoring, and evaluation, EGM, and research.

All team members are skillful in research methodologies, including literature search, data collection, and statistical analysis. The team has been trained by Campbell Collaboration and is therefore capable of carrying out the EGM processes like searching for studies, assessing their eligibility, critical quality appraisal of studies, and data extraction and coding.

**Information retrieval expertise**

The authors will be supported by Mr. Rodney Malesi, an experienced Senior Librarian affiliated with the United States International University, Kenya. Mr. Malesi is an astute librarian and expert in literature retrieval and has been involved in several systematic reviews and EGMs. Two research assistants, Edward Kusi Asafo‐Agyei and Clarice Panyin Nyan (a Ph.D. candidate at the University of Ghana) will assist with screening, coding, and quality appraisal of studies and papers to be included in the EGM under the daily supervision of Gloria Odei Obeng‐Amoako and Pacem Kotchofa. Gloria Odei Obeng‐Amoako will assist with the coordination of the daily EGM‐related activities to ensure smooth implementation of the protocol and the production of the EGM. Ashrita Saran will provide support on any methodological issues that may arise during the EGM production.

**Statistical analysis**

All the team members are skillful in statistical analysis useful for the proposed EGM production and the generation of the technical report that will accompany the EGM.

## DECLARATIONS OF INTEREST

The authors declare that they have no conflicts of interest. Though the BMGF team will be consulted for their perspectives on the design of the EGM, they will not be involved in the search for literature, screening, coding, and quality appraisal of the papers to be included in the EGM as well as the production of the EGM.

## SOURCES OF SUPPORT

### Internal sources

- New Source of support, Other

**External sources** 

- No sources of support provided

## Supporting information

Supporting information.
