# Supplementary material for: PROTOCOL: The impact of infrastructure on low income consumers' nutritious diet, women's economic empowerment, and gender equality in low‐and middle‐income countries: An evidence and gap map
Source: Campbell Syst Rev. 2023 Sep 5;19(3):e1353. doi: 10.1002/cl2.1353 (PMC10477950; doi:10.1002/cl2.1353)
Supplement: Supplementary file 1 — Supporting information. [file CL2-19-e1353-s001.docx]

Appendices

## 1 Definitions of some of sub-categories of infrastructure considered in the EGM

| Infrastructures | Platform |
| --- | --- |
| Irrigation systems | Irrigation system is an arrangement by which water is conveyed from a source to an area needing water to facilitate the production of desired crops (Kelly 1983).  Irrigation is the process to apply water to the soil to improve the crop growing, maintain landscapes and revegetate degraded soils in dry regions and periods of insufficient rainfall (Jimenez 2020).  Irrigation is the process of application of water to a land or soil. It is used for enhancing the growth of agricultural crops, maintaining landscapes, and revegetation of degraded soils in dry areas and periods of inadequate rainfall (Gavali 2016). |
| Wells and water wells | A well is a hole drilled into the ground to access water from an aquifer. A pipe and a pump are used to pull water out of the ground, and a screen filters out unwanted particles that could clog the pipe (Groundwater 2022). |
| Green infrastructure | Green infrastructure is an emerging planning and design concept that is principally structured by a hybrid hydrological/drainage network, complementing, and linking relict green areas with built infrastructure that provides ecological functions (Ahern 2007; Wright 2011) |
|  |  |
| Power supply (energy) | Indirect energy refers to the energy used to produce agricultural inputs. These inputs account for energy use that can be assigned to the agricultural sector but is used prior to reaching farms, including energy used in the production of fertilizers (raw materials, manufacturing, transport), production of pesticides (raw materials, manufacturing, transport), and production, storage, and transportation of seeding materials, among others  Direct energy refers to all energy inputs used directly in the agricultural production process; activities occurring on-farm and up to the farm gate (Paris 2022). |
| Storage and warehouses (sheds, cold rooms, pack houses) | Storage is an important marketing function, which involves holding and preserving goods from the time they are produced until they are needed for consumption. The storage of goods, therefore, from the time of production to the time of consumption, ensures a continuous flow of goods in the market. It protects the quality of perishable and semi-perishable products from deterioration; helps in the stabilization of prices by adjusting demand and supply. Storage also provides employment and income through price advantages (Agritech 2022). |
| Processing (grain mills) | Milling is the process of cleaning, tempering, and grinding cereal grains into flour and other milled grain products. Ground grain was one of civilization’s first foods (NAMA 2021). |
| Market stalls (spaces, lockups) | A market stall is a booth or stand where individuals or small businesses sell goods. These can range from artisan produce to food, antiques, clothes and much more. Markets can be in the form of a permanent fixture, as well as open-air markets, farmers markets, street markets and even car-boot sales (Square 2022). |
| Others (Slaughterhouses, landing sites, livestock vaccination parks) | A slaughterhouse or abattoir or meat works is a facility where animals are killed for consumption as food products. Slaughterhouses which process meat not intended for human consumption are sometimes referred to as Knacker’s yards or Knackeries (Definitions 2022).  Fish landing centers or sites are associated with small-scale marine and inland fisheries. They provide a location for first point of sale for products and provide a place where fishers can leave their boats and obtain supplies such as food, fuel and ice. The facilities, services and access to market vary (Ward 2022).  The vaccination of big animals such as cattle is carried out in vaccination parks set up by pastoralists, which allow to immobilize the animals so that the veterinary auxiliaries can carry out the vaccination. For small ruminants (sheep and goats), veterinary auxiliaries go from village to village to carry out the vaccinations (Tomarchio 2017). |
| Roads | Road infrastructure is understood to include all physical assets within the road reserve, including not only the road itself, but all associated furniture (signage etc), and all earthworks, drainage, structures (culverts, bridges, buildings etc)  Types of road: Murrum roads, gravel roads, earthen roads, kankar roads, bitumious road, concrete road  (Australian 1992). |
| Railways | Railroad infrastructure includes all the structures, buildings, land, and equipment to support rail lines. Railway infrastructure isn’t just limited to tracks; it includes all the structures, buildings, land, and equipment to support the rail lines. That support includes management, passenger, freight transport, and maintenance ( Constructor 2021).  Types of railways: surface railways, elevated railways, underground railways (Ferrovial 2022). |
| Bridges | A bridge is to allow people or cargo easy passage over an obstacle by providing a route that would otherwise be uneven or impossible (Law 2022). |
| Information centers | Information center is a public space where community members have shared public access to ICT and use it to implement social development programs, support the social and personal development of the individuals, communities and contribute to improving the quality of life of community people(Heeks 2002). |
| Radio stations | The radio station subsystem (RSS) is the physical equipment that provides coverage to prescribed geographical areas, known as cells. It contains equipment required to communicate with the user equipment. Functionally, an RSS consists of a control function performed by the base station controller (BSC) and a transmitting/receiving function carried out by the base station transceiver (BTS) system. The BTS is the radio transmission/receiving equipment and covers a cell. An RSS can serve several cells and can have multiple base station transceivers (Garg 2010). |
| Telecommunication masts to facilitate and enhance communication | A telecommunication mast is a freestanding structure which supports antennas at a height where they can transmit and receive radio waves (Bello 2010). |

## 2 List of potential organizational websites and databases for grey literature and hand search for EGM

CGIAR research library: IFPRI, Biodiversity, World Agroforestry, and International Livestock Research Institute, DfID Research for Development Outputs, British Library for Development Studies (BLDS), FAO AGRIS, IMMANA grant database, The 3ie impact evaluation database, Innovation Poverty Action (IPA) and The Abdul Latif Jameel Poverty Action Lab (J-PAL), Campbell Collaboration, Campbell South Asia, Cochrane library for reviews and trials, The World Bank IEG evaluations, USAID Development Experience Clearinghouse, Agriculture, Nutrition and Health (ANH) Academy conference, OECD/DAC Evaluation database, The World Bank Economic Review, Google Scholar, Bill and Melinda Gate Foundation (BMGF), International Water Management Institute (IWMI) World Bank, International Fund for Agricultural Development (IFAD), United Nations Women, United Nations Food and Agriculture Organization (FAO), International Food Policy Research Institute (IFPRI) and the Alliance for a Green Revolution in Africa (AGRA), International Institute for Environment and Development (IIED), AgriProFocus, Donor Committee for Enterprise Development (DCED), Swiss Agency for Development and Cooperation and United Nations Office for Project Services (UNOPS),OpenGrey, Networked Digital Library of Theses and Dissertations (NDLTD) (www.theses.org)/ National Bureau of Economic Research (NBER) and EBSCO- OpenDissertations

## 3 Examples of search terms for searching of studies/papers New Appendix

The following are some of the examples of search terms we will apply in searching for published studies or reports in the identified academic electronic databases:

## Population search terms

### LMIC _1

Afghanistan OR angola OR bangladesh OR benin OR dahomey OR “benin republic” OR botswana OR “burkina faso” OR “burkina fasso” OR “upper volta” OR burundi OR urundi OR Bhutan OR “cabo verde” OR “cape verde” OR “cameroon OR cameron OR cameroun OR “central african republic” OR “ubangi shari” OR chad OR tchad OR comoros OR “comoro islands” OR “iles comores” OR congo OR zaire OR “cote d ivoire” OR “cote divoire” OR “cote d ivoire” OR “ivory coast” OR djibouti OR “french somaliland” OR “Equatorial Guinea” OR eritrea OR ethiopia OR Eswatini OR gabon OR “gabonese republic” OR gambia OR ghana OR “gold coast” OR guinea OR india OR iran OR kenya OR lesotho OR basutoland OR liberia OR madagascar OR “malagasy republic” OR malawi OR nyasaland OR mali OR maldives OR mauritania OR maritius OR mozambique OR namibia OR nepal OR niger OR nigeria OR rwanda OR ruanda OR (“sao tome and principe”) OR senegal OR Seychelles OR “Sierra Leone” OR somalia OR “south africa” OR “south sudan” OR “sri lanka” OR pakistan OR tanzania OR tanganyika OR togo OR “togolese republic” OR uganda OR zambia OR zimbabwe OR “northern rhodesia” OR “global south” OR “africa south of the sahara” OR “sub-saharan africa” OR “subsaharan africa” OR “africa, central” OR “central africa” OR “africa” OR sahara OR “africa, southern” OR “southern africa” OR “africa, eastern” OR “east africa” OR “eastern africa” OR “africa, western” OR “west africa” OR “western africa” OR “asia” OR “asia, southern” OR “southern asia” OR “south asia”

### LMIC _2

“developing country” OR “developing countries” OR “developing nation*“ OR “developing population*“ OR “developing world” OR “less developed countr*“ OR “less developed nation*“ OR “less developed population*“ OR “less developed world” OR “under developed nation*“ OR “under developed population*“ OR “under developed world” OR “underdeveloped countr*“ OR “underdeveloped nation*“ OR “underdeveloped population*“ OR “underdeveloped world” OR “middle income countr*“ OR “middle income nation*“ OR “middle income population*“ OR “low income countr*“ OR “low income nation*“ OR “low income population*“ OR “lower income countr*“ OR “low-income countries” OR “middle income countries” OR “lower income nation*“ OR “lower income population*“ OR “developing countries” OR “developing nation” OR “underdeveloped nation” OR “third world countries” OR “emerging economies” OR “poor countr*“ OR “poor nation*“ OR “poor population*“ OR “poor world” OR “poorer countr*“ OR “poorer nation*“ OR “poorer population*“ OR “poorer world” OR “developing econom*“

### LIC

“low-income consumer” OR “low-income consumers” OR “low income consumer” OR “low-income consume*“ OR “low income consume*“ OR “food poor*“ OR “below poverty line” OR “disadvantaged people” OR “low income people” OR “vulnerable people” OR OR “low income population” OR “vulnerable population” OR “underprivileged population” OR ““smallholder farmer*“ OR “low stakeholder*“ OR “low spending people” OR “low bargaining power” OR “slum” OR “rural areas” OR “remote areas” OR “isolated” OR “disempowered...” OR “subsistence” OR “low-income consumers” OR “low income” OR “low pay” OR “poverty”

## Intervention search terms

### Infrastructure

“Agricultural infrastructure” OR “agriculture infrastructure” OR agriculture OR agricult* OR infrastructure OR infrastruct* OR “agricultural technolog*” OR “agricultural facilities” OR “agricultural facility” OR “facility “or “Productive Infrastructure” OR “Rural Infrastructure” OR “Large Infrastructure”

### Production

“irrigation system*“ OR irrigation OR “water well*“ OR “green infrastructure*“ OR “garden* infrastructure*“ OR “Electri* infrastructure” OR “power supply” OR “energy supply” OR solar OR “solar energy” OR “solar power” OR “solar power irrigation” OR “solar energy irrigation” OR “green infrastructures” OR “green revolution infrastructures” OR “agricultural mechanization” OR “agricultural machiner*” OR “pastoral* infrastructure” OR “agropastoral infrastructure” OR “agro-pastoral infrastructure” OR “ejido” OR “silvopastoral infrastructure” OR “farm infrastructure*“ OR “producer*“ OR “grower*“ OR “agronomy infrastructure” OR “husbandry infrastructure” OR “aquacultur* infrastructure” OR “floricultur* infrastructure” OR “horticultur* infrastructure” OR “cultivat* infrastructure” OR “dairy infrastructure” OR “livestock infrastructure” OR “crop* infrastructure”

### Post Production

“Food Handling” OR “Food Storage” OR “Food Quality” OR “storage facilit*“ OR storage OR storehouse OR store OR Warehouse* OR sheds OR “cold rooms” OR coldroom OR “processing facilities” OR process* OR processing OR mill OR “grain mills” OR market OR space* OR “market place” OR “market stalls” OR booth OR “market lockups” OR toilets OR lavatory OR bathroom OR sanitation OR hygiene OR “sanitary facilities” OR “market sanitation” OR market place toilet” OR “market* toilets” OR slaughterhouse* OR abattoir OR butcher* OR “landing site*” OR “fishing sites” OR livestock OR “vaccination parks” OR vaccination OR “animal vaccination park”

## Distribution

“road*“ OR “bridge*“ OR “railway*“ OR “transportation” OR transport* OR “transportation infrastructure*“ OR “emergency transportation” OR “sustainable transportation” OR “rural road*“ OR “distribution infrastructure”

### Information

‘’information” OR ‘’information centers” OR telecommunication OR “telecommunication masts” OR mast OR “information cent*“ OR “telecommunication masts” OR “information infrastructure” OR “telecommunication infrastructure*”

## Outcomes

### Nutritious diets

“nutritious diets’ OR “healthy food” OR “Healthy diets” OR “healthy meal*“ OR “Diet quality” OR “diet adequacy” OR “dietary diversity” OR “nutrition security” OR “food availability” OR “food affordability index” OR “food accessibility” OR OR “food agency” OR “nutrit* food diet” OR diet OR food OR “meal*“ OR “Per capita dietary energy supply” OR DES OR “Food Insecurity Experience Scale” OR FIES OR “Household Food Insecurity Access Scale” OR HFIAS OR “Minimum Acceptable Diet” OR MAD OR “Minimum Dietary Diversity” OR MDD OR “Minimum Dietary Diversity for Women” OR MDD-W OR “Mean adequacy ratio” OR MAR OR “food price” OR “food price index” OR “fresh food” OR “fresh food retail volume” OR “market level food diversity score” OR “food price volatility” OR “income variation in food access” OR “Weather seasonality indexes in agriculture” OR “Women empowerment in nutrition index” OR “WENI”

### Women’s economic empowerment

“Women economic empowerment” OR “Women empowerment” OR “The Five Domains of Empowerment” OR “5DE” OR “agricultural production” OR “Input in production decisions” OR “autonomy in production” OR “access and control over productive resources” OR “control over productive resources” OR “ productive resources access” OR “assets ownership” OR “ownership of assets” OR “purchase of assets” OR “sale of assets” OR “transfer of assets” OR “access to credit” OR “decisions about credit” *OR* “time use” OR “time allocation” OR “leisure” OR “workload” OR “resource allocation” OR “women choices” OR “WEAI” OR “WENI” OR “WELI” OR “A-WEAI” OR “Pro- WEAI” OR “women voices” OR “wages” OR “income” OR “women leadership”

### Gender equality

“Gender Equity” OR “gender equality” OR “gender equalit*” OR “gender equal* “OR “gender parity index” OR “gender inequality” OR “gender inequity” OR “gender disparity” OR “ gender norm” OR “gendered institution” OR “gender mainstreaming” OR “gender” OR “women” OR “men” OR “boys” OR gender stereo*“ OR “gender norm*“ OR “gender role*“ OR “gender equit*“ OR “gender inequit* OR “gender inequal***”** OR “cultural and gender norms”

## Study Design

- “Impact Evaluation” or “summative Evaluations” or ‘Final Evaluation” OR “Process Evaluation’ or “Retrospective Evaluation” OR, Thematic Evaluations; or report or descriptive or explanatory or narrative
- OR “Systematic Reviews” or “Meta-Evaluations” or “Meta Analysis” or “Modelling studies” OR “Literature Reviews”
- (random* or experiment* or (match* adj2 (propensity or coarsened or covariate)) or “propensity score” or (“difference in difference*“ or “difference-in-difference*“ or “differences in difference*“ or “differences-in-difference*“ or “double difference*“) or (“quasi-experimental” or “quasi experimental” or “quasi-experiment” or “quasi experiment”) or ((estimator or counterfactual) and evaluation*) or “instrumental variable*“ or (IV adj2 (estimation or approach)) or regression discontinuity or time series or segment* regression).
- OR heckmann; “quantitativ* synthes*” OR “mixed method*“ OR “mixed‐method*” OR “quantitativ* synthes*” OR “mixed method*“ OR “mixed‐method*” OR “quantitativ* synthes*” OR “mixedmethod*“ OR “mixed‐method*” OR “qualitative method*”
- “meta regression” OR “meta synth*“ OR “meta‐synth*“ OR “metaanaly*“ OR metaanaly* OR meta‐analy* OR metanaly* OR “metaregres-sion” OR meta‐regression OR “methodologic* overview” OR “pool* ana-lys*“ OR “pool* data” OR “quantitative* overview” OR “researchintegration” OR “meta regression” OR “meta synth*“ OR “meta‐synth*“ OR “meta analy*“ OR metaanaly* OR meta‐analy* OR metanaly* OR “metaregression” OR meta‐regression OR “methodologic* overview” OR “pool* analys*“ OR “pool* data” OR “quantitative* overview” OR “re-search integration” OR “meta regression” OR “meta synth*“ OR “meta‐synth*“ OR “meta analy*“ OR metaanaly* OR meta‐analy* OR me-tanaly* OR “metaregression” OR meta‐regression OR “methodologic*overview” OR “pool* analys*“ OR “pool* data” OR “quantitative* over-view”

## 4 Coding form

| **Parent code** | **Child code** | **Grandchild code** |
| --- | --- | --- |
| **Population** | ***Central Africa*** | Angola, Burundi, The Central African Republic  Chad, Democratic Republic of Congo, Congo, Rwanda |
|  | ***Eastern Africa*** | Comoros, Eritrea, Ethiopia, Kenya, Madagascar, Somalia, South Sudan, Sudan, Tanzania, Uganda |
|  | ***Southern Africa*** | Comoros, Eritrea, Ethiopia, Kenya, Madagascar, Somalia, SouthSudan, Sudan, Tanzania, Uganda |
|  | ***Western Africa*** | Benin, Burkina Faso, Cape Verde, Cameroon, Côte d’Ivoire, Equatorial Guinea, Gabon, The Gambia, Ghana, Guinea, Guinea-Bissau, Liberia, Mali, Mauritania, Niger, Nigeria, Sao Tome and Principe, Senegal, Sierra Leone, Togo |
|  | ***South Asia*** | Afghanistan, Bangladesh, Bhutan, Iran, India, Pakistan, Sri Lanka, Maldives |
| **Interventions** | **Production** | Irrigation systems and water wells; Gardens and other green infrastructure; On-farm energy and power supply; other agricultural and farm |
|  | **Post-production** | Market facilities; Storage facilities processing facilities; livestock facilities;, On-farm Energy and power supply |
|  | **Distribution** | Roads, Railways, Bridges |
|  | **Information** | Information centres, Telecommunication masts, radio stations |
| **Outcomes** | **Nutritious diets** | Food availability: farm level, food Availability: market level food, food accessibility: affordability, food accessibility: presence of food market, food consumption, diet quality: individual dietary diversity, diet quality: household dietary diversity, sociocultural dimensions of foods |
|  | **Women’s economic empowerment** | Agricultural production, access and control over productive resources, income, time, leadership, Gender Parity Index (GPI) |
|  | **Gender equality** | Economic opportunities and outcomes; social outcomes; leadership, agency, and collective action, reduced exposure to risk |
| Study design | **Primary studies** | Experiment, quasi-experiment (including natural experiment), non -experimental, qualitative studies |
|  | **Reviews** | Systematic reviews, meta-analyses, other reviews |
| **Types of evaluation** | **Impact evaluation, formative evaluation, process evaluation** |  |
| **Bibliographic information** | **Publication status** | Published, completed, and unpublished, ongoing |
|  | **Publication type** | Peer review articles, preprint-peer review articles, policy briefs, reports, working papers, conference papers, book chapter |
|  | **Date of publication** | 2000-2022 |

[Enter text here]
